# Mapping Inflammatory Markers in Cerebrospinal Fluid Following Aneurysmal Subarachnoid Hemorrhage: An Age- and Sex-Matched Analysis †

**DOI:** 10.3390/ijms26031302

**Published:** 2025-02-03

**Authors:** Katharina Sophie Seyfried, Benedikt Kremer, Catharina Conzen-Dilger, Michael Veldeman, Ulf Bertram, Christian Blume, Christian Andreas Mueller, Tianshu Bi, Kerstin Jütten, Hans Clusmann, Anke Höllig

**Affiliations:** Department of Neurosurgery, Uniklinik RWTH Aachen, 52074 Aachen, Germany; bkremer@ukaachen.de (B.K.); cconzen@ukaachen.de (C.C.-D.); mveldeman@ukaachen.de (M.V.); ubertram@ukaachen.de (U.B.); cblume@ukaachen.de (C.B.); camueller@ukaachen.de (C.A.M.); tbi@ukaachen.de (T.B.); kjuetten@ukaachen.de (K.J.); hclusmann@ukaachen.de (H.C.); ahoellig@ukaachen.de (A.H.)

**Keywords:** subarachnoid hemorrhage, neuroinflammation, cerebrospinal fluid, cytokines, chemokines

## Abstract

Despite extensive research on aneurysm treatment and neurocritical care, aneurysmal subarachnoid hemorrhage (SAH) is still a life-threatening disease, often leaving survivors with lasting neurological and cognitive impairments. Early brain injury (EBI) and delayed cerebral ischemia (DCI) are the main contributors to brain damage, with neuroinflammation being a critical shared pathophysiological process. While numerous inflammatory markers and their temporal profiles in cerebrospinal fluid (CSF) have already been identified, comparisons with age- and sex-matched controls are limited. This study analyzed CSF from 17 SAH patients requiring an external ventricular drain (EVD) due to symptomatic hydrocephalus, sampled on days 4 and 10 post-ictus. An age- and sex-matched control group included 17 cerebrovascularly healthy patients requiring lumbar drains during aortic surgery. Chemokines and cytokines were quantified using immunoassays. Significantly elevated markers in SAH patients across both time points included MCP-1, CXCL-13, Eotaxin-1, CXCL-10, IL-8, and MIF. MIP-1α and MIP-1β showed significant differences at particular time points, indicating a distinct temporal profile for each parameter. These findings highlight neuroinflammation’s key role in intracranial and systemic pathophysiology following SAH, emphasizing its complexity and individual variability. Knowing demographic factors impact the specific manifestations of pathophysiological processes, the comparison with an age- and sex-matched control group is meaningful.

## 1. Introduction

Despite all efforts made in recent years to improve aneurysm treatment and neurocritical care, aneurysmal subarachnoid hemorrhage (SAH) remains a life-threatening disease [1,2]. Moreover, even SAH patients with formally good outcomes without any remaining gross neurological deficits often suffer from long-term neurocognitive impairment [3]. There is a huge proportion of SAH patients who never return to work and suffer from lifelong cognitive deficits, which lead to reduced quality of life [4]. Furthermore, there is a high risk of developing post-stroke dementia after SAH [5,6].

Over the last decades, two main contributors to brain damage after SAH have been identified: early brain injury (EBI) and delayed cerebral ischemia (DCI). EBI refers to pathophysiological processes that develop during the first 72 h after the ictus, such as microcirculatory dysfunction, blood–brain barrier breakdown, neuroinflammation, cerebral oedema, oxidative cascades, and neuronal death [7]. DCI typically occurs between the 4th and the 10th day after the ictus and is defined by a new neurological deficit or cerebral infarction diagnosed by imaging or autopsy that cannot be attributed to other causes [2,8].

There are multiple mechanisms involved in DCI, such as (micro)vascular dysfunction, microthrombosis, impairment of the glymphatic system, failure of autoregulation, inflammatory processes, and neuroelectric disturbances [9].

Neuroinflammatory processes are well known contributors to EBI as well as DCI following SAH, and a correlation between serum and cerebrospinal fluid (CSF) inflammatory markers and clinical outcome has been shown in many studies [10,11,12,13,14]. Furthermore, there is evidence suggesting that inflammatory processes may also play a causal role in the growth and rupture of intracranial aneurysms [15].

For many inflammation-related proteins, a characteristic temporal profile following SAH has already been identified, allowing for a better understanding of the time course of inflammatory processes, especially of central nervous system (CNS)-specific inflammation after SAH [16,17]. Recent studies have highlighted the future potential of targeting neuroinflammation pharmacologically [18,19].

However, the inflammatory system is complex, with countless interactions. Thus, the identification of a single biomarker or therapeutic target within the neuroinflammatory system is nearly utopian. To address this complexity, broader analyses of inflammatory proteins in CSF, including the comparison with appropriate age- and sex-matched healthy controls, are required. Therefore, an analysis of a broad panel of different chemokines and cytokines in the CSF of SAH patients compared with an age- and sex-matched cohort of cerebrovascularly healthy controls was conducted.

## 2. Results

### 2.1. Demographic Characteristics

The patient population as well as the control group included fourteen women and three men. Median age was 56 years (95% confidence interval (CI): 49–67 years) in the SAH group and 65 years (95% CI: 58–69 years) in the control group. Exact age matching was not always possible, which accounts for the difference in medians. The maximum deviation between matched pairs was 26 years. There was no significant difference in age between the groups (*p* = 0.0829).

Ten patients were in good clinical condition upon arrival and had a Hunt and Hess score between 1 and 3, while seven patients had a Hunt and Hess score of 4 or 5.

Using the World Federation of Neurosurgical Societies score (WFNS), five patients had a score of I–III, and 12 patients had a score of IV–V.

Regarding radiological features, thirteen patients suffered from an additional intracerebral or intraventricular hemorrhage, and four patients had a thick subarachnoid blood clot. There were no patients with a modified Fisher score of 1 or 2.

Thirteen aneurysms were in the anterior circulation. Most aneurysms (*n* = 13) were treated endovascularly, while only four aneurysms were clipped surgically. Seven patients suffered from delayed cerebral ischemia. Six patients required placement of a ventriculoperitoneal shunt due to posthemorrhagic hydrocephalus.

At time of discharge, seven patients reached a favorable clinical outcome, defined as a Glasgow Outcome Scale (GOS) score of 4 or 5. One patient died during the hospital stay, and nine patients reached an unfavorable outcome (GOS 1 to 3). Four patients were lost to follow-up before the six-month outcome could be determined. One patient died during the follow-up period; another one deteriorated from GOS 5 to 2. No patient with an unfavorable outcome recovered to reach a favorable outcome.

Demographic characteristics are summarized in Table 1. For a detailed visualization of age- and sex-matched pairs of SAH patients and controls please see Supplementary Table S3.

### 2.2. Inflammatory Protein Profiles in CSF of SAH Patients vs. Healthy Controls

#### 2.2.1. Comparative Analysis of the Number of Measurable Values

For some parameters (Interleukin-1-receptor antagonist (IL-1-RA), Interleukin-6 (IL-6), Interleukin-17a (IL-17a), Growth-regulated protein alpha (GRO-α)/C-X-C motif chemokine ligand 1 (CXCL1), Interleukin-1α (IL-1α), Interleukin-10 (IL-10), Interleukin-21 (IL-21), and Interleukin-9 (IL-9), only a few concentrations exceeded the detection limit, particularly in the control group, so that the number of detectable values was compared between the groups.

However, no statistically significant differences could be detected for either day 4 or day 10 after SAH. A summary of the data is illustrated in Supplementary Figure S1.

#### 2.2.2. Direct Comparisons of Absolute Protein Levels

If more than 50% of the values were available, absolute protein levels were compared rather than the number of measurable values. For Monocyte Chemoattractant Protein-1 (MCP-1)/C–C motif chemokine ligand 2 (CCL2), median values were statistically significant higher in SAH patients compared to healthy controls (1014 pg/mL in controls vs. 2100 pg/mL on day 4 (*p* = 0.0002) and 2534 pg/mL on day 10 (*p* = 0.001)).

**Figure 1:**
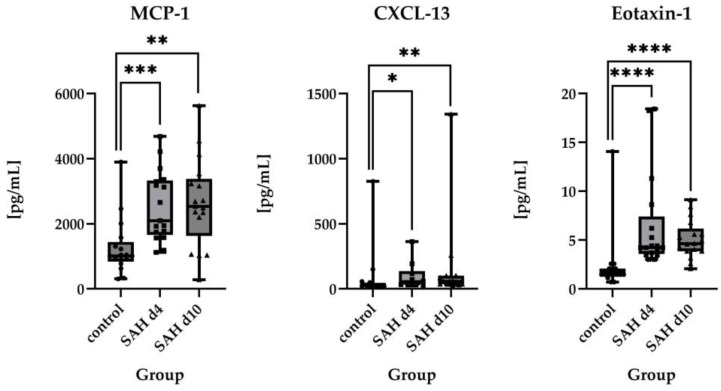

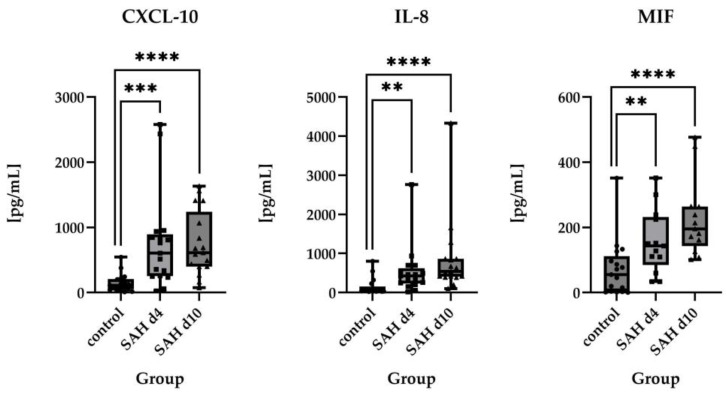
Comparison of absolute values of MCP-1 (control *n* = 17, SAH d4 *n* = 17, SAH d10 *n* = 17), CXCL-13 (control *n* = 16, SAH d4 *n* = 10, SAH d10 *n* = 14), Eotaxin-1 (control *n* = 16, SAH d4 *n* = 17, SAH d10 *n* = 17), CXCL-10 (control *n* = 17, SAH d4 *n* = 17, SAH d10 *n* = 17), IL-8 (control *n* = 17, SAH d4 *n* = 17, SAH d10 *n* = 17), and MIF (control *n* = 17, SAH d4 *n* = 13, SAH d10 *n* = 13) in healthy controls vs. SAH patients on day 4 and day 10 after ictus, displayed as individual values. All these parameters showed statistically significant differences between control and SAH patients at both time points. Statistical significance is indicated as follows: *p* < 0.0001: extremely significant; **** *p* = 0.0001 to 0.001: extremely significant; *** *p* = 0.001 to 0.01: very significant; ** *p* = 0.01 to 0.05: significant; * *p* ≥ 0.05: not significant: ns.

The same applied to C-X-C motif ligand 13 (CXCL-13) (28.55 pg/mL in controls vs. 54.46 pg/mL on day 4 (*p* = 0.0174) and 56.34 pg/mL on day 10 (*p* = 0.0031)), Eotaxin-1 (CC-chemokine ligand 11/CCL11) (1.480 pg/mL in controls vs. 4.230 pg/mL on day 4 (*p* < 0.0001) and 4.640 pg/mL on day 10 (*p* < 0.0001)), IFN-gamma-inducible protein 10 (IP-10)/C-X-C motif chemokine ligand 10 (CXCL-10) (110.7 pg/mL in controls vs. 603.3 pg/mL on day 4 (*p* = 0.0001) and 611.7 pg/mL on day 10 (*p* < 0.0001)), and Interleukin 8 (IL-8)/C-X-C motif chemokine ligand 8 (CXCL8) (54.74 pg/mL in controls vs. 425.5 pg/mL on day 4 (*p* = 0.0015) and 541.6 pg/mL on day 10 (*p* < 0.0001)). For macrophage migration inhibitory factor (MIF), a statistically significant difference between the groups could also be detected on both time points after the ictus (55.17 pg/mL in controls vs. 143.4 pg/mL on day 4 (*p* = 0.0060) and 195.6 pg/mL on day 10 (*p* < 0.0001)). For a visual representation of the time course of these parameters, please see Figure 1.

**Figure 2:**
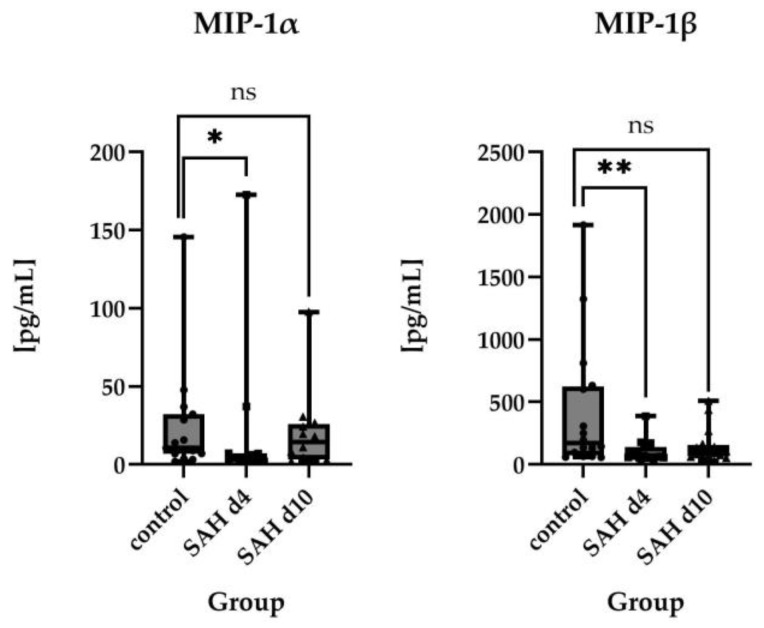
Comparison of absolute values of MIP-1α (control *n* = 15, SAH d4 *n* = 12, SAH d10 *n* = 12) and MIP-1β (control *n* = 16, SAH d4 *n* = 17, SAH d10 *n* = 16) in healthy controls vs. SAH patients on day 4 and day 10 after ictus, displayed as individual values. For MIP-1α, a significantly lower median was observed on day 4 after ictus compared to controls, while levels increased by day 10. MIP-1β showed a similar time course as MIP-1α and decreased on day 4 after SAH, while levels rose on day 10 after SAH. Statistical significance is indicated as follows: *p* < 0.0001: extremely significant; ** *p* = 0.01 to 0.05: significant; * *p* ≥ 0.05: not significant: ns.

For macrophage inflammatory protein-1α (MIP-1α/C–C motif chemokine ligand 3 (CCL3)), a significantly lower median was observed on day 4 after ictus compared to controls (3.635 pg/mL vs. 10.59 pg/mL, *p* = 0.0454). Interestingly, levels increased by day 10, exceeding control values (14.45 pg/mL), though this difference was not statistically significant. The same applied for macrophage inflammatory protein-1β (MIP-1β)/C–C motif chemokine ligand 4 (CCL4) (171.7 pg/mL in controls vs. 67.25 pg/mL on day 4 (*p* = 0.0077) and 98.19 pg/mL on day 10 (*p* = 0.110)). For a visual representation of the time course of these parameters, please see Figure 2.

No statistically significant differences were found between the groups for stromal cell-derived factor 1α (SDF-1 α)/C-X-C motif chemokine ligand 12 (CXCL12), Interleukin-2 (IL-2), and triggering receptor expressed on myeloid cells-2 (TREM-2).

### 2.3. Time Course of Inflammatory Proteins in CSF After SAH

No statistically significant differences were found between day 4 and day 10 post-ictus for the examined proteins. However, Eotaxin-1, IL-1-RA, IL-6, IL-10, and IL-21 showed a tendency to return to baseline levels (assumed as the levels of the control group) over time.

In contrast, MCP-1, CXCL-13, IL-8, MIF, SDF-1α, IL-2, TREM2, GRO-α, IL-1α, IL-17a, CXCL-10, and IL-9 appeared to deviate further from control values as time progressed.

**Figure 3:**
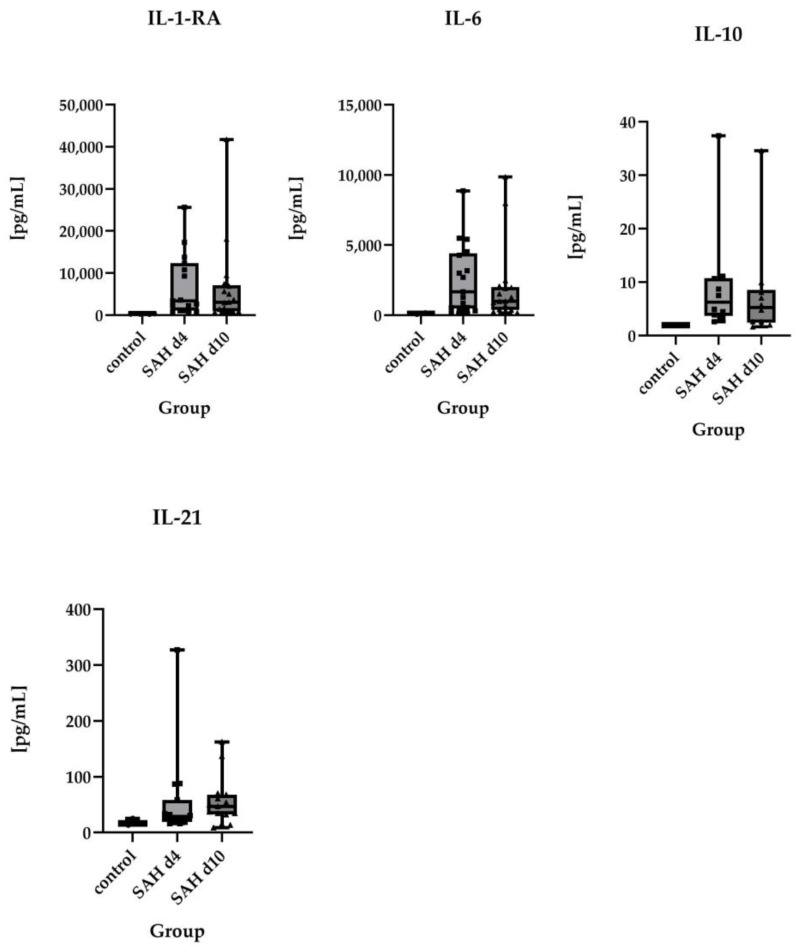
Comparison of absolute values of IL-1-RA (control *n* = 6, SAH d4 *n* = 15, SAH d10 *n* = 17), IL-6 (control *n* = 2, SAH d4 *n* = 17, SAH d10 *n* = 17), IL-10 (control *n* = 1, SAH d4 *n* = 10, SAH d10 *n* = 10), and IL-21 (control *n* = 3, SAH d4 *n* = 15, SAH d10 *n* = 15) in healthy controls vs. SAH patients on day 4 and day 10 after ictus, displayed as individual values. These parameters were rarely above the detection limit in healthy controls, making a comparison between control and SAH patients difficult. Consequently, no statistically significant differences between healthy controls and SAH patients were found, but all these parameters showed distinct temporal profiles after SAH. However, there were no statistically significant differences between SAH patients on day 4 and day 10 after the ictus. Due to the lack of statistically significant differences between the groups, symbols of significance were omitted in order to make the graphs clearer.

**Figure 4:**
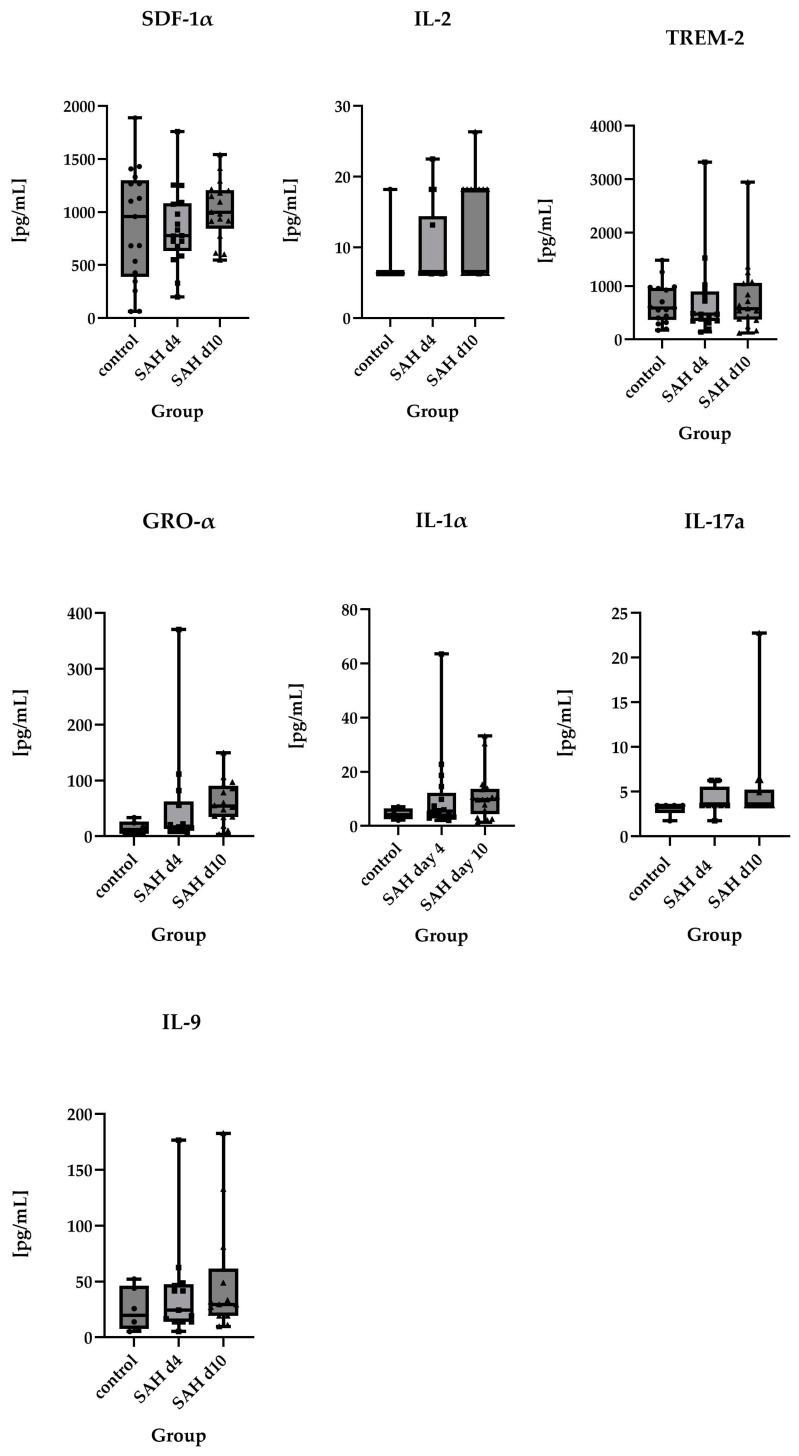
Comparison of absolute values of SDF-1α (control *n* = 17, SAH d4 *n* = 17, SAH d10 *n* = 17), IL-2 (control *n* = 11, SAH d4 *n* = 14, SAH d10 *n* = 15), TREM-2 (control *n* = 17, SAH d4 *n* = 16, SAH d10 *n* = 17), Gro-α (control *n* = 6, SAH d4 *n* = 14, SAH d10 *n* = 16), IL-1α (control *n* = 4, SAH d4 *n* = 17, SAH d10 *n* = 17), IL-17a (control *n* = 5, SAH d4 *n* = 8, SAH d10 *n* = 14), and IL-9 (control *n* = 6, SAH d4 *n* = 13, SAH d10 *n* = 15) in healthy controls vs. SAH patients on day 4 and day 10 after ictus, displayed as individual values. There were also no statistically significant differences between controls and SAH patients or between different time points after SAH. However, these parameters showed a tendency to deviate further from baseline levels after SAH. Due to the lack of statistically significant differences between the groups, symbols of significance were omitted in order to make the graphs clearer.

Interestingly, MIP-1α levels decreased on day 4 after SAH to rise again and exceed control values on day 10 after SAH. MIP-1β also decreased after SAH and increased on day 10 but stayed below the control levels. For a visual representation of the time course of inflammatory proteins in CSF after SAH please see Figure 3 and Figure 4.

Nevertheless, none of these observations reached statistical significance. For visual representation of the time course of the parameters shown in the previous section, please review Figure 1 and Figure 2.

### 2.4. Subgroup Analysis

Subgroup analysis was carried out for different demographic and clinical characteristic. However, no statistically significant influences of any of these factors could be found. For a detailed overview please see the Supplementary Materials.

## 3. Discussion

### 3.1. Inflammatory Protein Profiles and Their Temporal Pattern in CSF of SAH Patients vs. Healthy Controls

Neuroinflammation is a key mechanism in both EBI and DCI, contributing significantly to neuronal damage and lasting neurological deficits in SAH patients [20]. Furthermore, the inflammatory reaction following SAH extends beyond the CNS, as systemic inflammation can drive extracranial complications [21]. However, peak inflammation still occurs in the CNS [17]. Our data emphasize the relevance of neuroinflammation and demonstrate its progression in a cohort with a stable case number compared to age- and sex-matched controls.

Neuroinflammation (or better its imbalance) is the common denominator of numerous pathologies of the CNS and their chronic consequences. Probably one of the most studied pathologies resulting in a long-lasting neuroinflammatory response is traumatic brain injury (TBI). From TBI research, we know that reactive neuroinflammation is complex, involving cellular and protein-related responses, among others [22,23,24]. Persistent neuroinflammation may be linked to secondary pathologies, such as the development of dementia and other neurodegenerative diseases [24,25]. Thus, neuroinflammation may represent a major therapeutic target, as neither TBI nor SAH can be reversed.

In relation to SAH, individual parameters have often been investigated for their prognostic value concerning outcomes or the occurrence of secondary complications. Most of these analyses are limited to a small spectrum of inflammatory parameters, and the sample size is low.

The most frequently studied inflammatory response after SAH is certainly the increase in IL-6, which can be observed in blood and CSF. IL-6 is a pleiotropic cytokine, and its increase is an unspecific reaction induced by various triggers. Therefore, its utility as a biomarker is uncertain [26]. Similar to other studies [26,27], the variance in IL-6 levels is large. The severity of the disease as well as concomitant factors such as infectious diseases may influence the rise in IL-6 levels. Furthermore, the increase in IL-6 levels is just one piece of the puzzle in the network of inflammatory reactions following SAH.

In 2016, Niwa et al. [28] compared several CSF cytokine and chemokine levels following SAH (*n* = 10 patients) with samples from five patients with unruptured aneurysms using a similar approach as in our study. They found a significant increase in the concentration of IL-6 after SAH as well as a correlation between high IL-6 levels and unfavorable clinical outcomes. However, due to the small sample size, data with respect to outcome are uncertain.

Niwa and colleagues also observed a rise in MCP-1 after SAH with a peak on day 3 and a subsequent decrease. Nonetheless, we observed an ongoing rise in MCP-1 during our study. These differences may be due to the low sample sizes of both studies as well as sampling biases, as Niwa and colleagues predominantly included SAH patients with a good Hunt and Hess score (6 out of 10: Hunt and Hess 2). Furthermore, the specific immunoassays used differed, and CSF was drawn from the basal cistern during the clipping procedure, whereas in our study, CSF from an external ventricular drain (EVD) was used. Other cytokine patterns are similar to those we measured; CXCL-10 (IP-10, respectively) showed a peak after the ictus with a following decrease, which is well in line with our results [28].

Two studies from the group of Vlachogiannis and colleagues examined cytokine levels following SAH, too. For CXCL-10 and IL-6, a somewhat longer-lasting increase in values was seen [13,16]. However, both studies had relatively small sample sizes, making it difficult to draw general conclusions about the normal time course of these parameters.

A parameter that has been extensively studied in the context of ischemic stroke is IL-1-RA [29]. In a study by Gruber et al., a peak in IL-1-RA in CSF was observed on day 6 after SAH with a subsequent decrease in good-grade patients and persistent elevated concentrations in poor-grade patients [30]. This was consistent with our results, as we saw decreasing IL-1-RA concentrations on day 10 after SAH. Analyzing our data, control levels were below the detection limit in most subjects, making meaningful comparison between groups difficult.

Other studies focused on the cellular immunoreaction following SAH, specifically Treg/TH17 balance [31]. However, in this project, various cytokines (IL-17a, IL-6, IL-10 and IL-2) were measured, too, and were shown to be increased in serum and CSF of severely impacted SAH patients compared to healthy controls [31]. In our study, IL-17a was below the detection threshold in more than 50% of cases in the control group, so a meaningful statistical analysis was not possible. This might be due to our less severely impacted study population.

TREM2 is a protein linked to cellular inflammation, as it is involved in the modulation of the microglial neuroinflammatory responses. Following experimental SAH, a protective effect on neuronal cell death could be shown [32]. However, human studies are still lacking, and our data on TREM2 are not conclusive and probably hampered by the low sample size.

For several of the parameters measured here, their relevance has not yet been clearly established, as there is currently no comparative data available in the context of SAH. We are referring here to the factors CXCL-13, IL-1α, IL-21, SDF-1α, MIP-1α, MIP-1β, and IL-9.

Even though our data are limited due to the small sample size, our results highlight two essential aspects of reactive neuroinflammation following SAH. First, we see that a wide range of inflammatory parameters respond after the event, so interactions are highly probable, and the effect cannot be attributed to a single pathway. Second, the interindividual variability is remarkably high. Whether the individual extent of neuroinflammation has prognostic value remains to be determined.

In summary, several studies have already demonstrated changes in inflammatory parameters following SAH. Al-Tamimi and colleagues highlighted a drastic increase in inflammatory mediator levels in CSF following SAH compared to an age-matched control group [17]. Similarly, Niwa and colleagues demonstrated a dynamic increase in cytokines and chemokines in the CSF following SAH compared to samples from patients with unruptured aneurysms [28].

With our study, we add further data on the neuroinflammatory pattern following SAH by further expanding the range of measured parameters, maintaining a stable number of patients over time, and providing results from an age- and sex-matched control group.

### 3.2. Limitations of the Study

One major limitation of this study is the small sample size. 

Additionally, the study population is quite heterogenous regarding clinical severity, as some SAH patients were only mildly impacted while others were severely ill and suffered from permanent neurological damage. However, in terms of aneurysm location and treatment, greater heterogeneity would be preferable as most aneurysms were located in the anterior circulation and treated endovascularly. A larger, more diverse study population would be desirable to confirm our results. 

Moreover, many proteins had a limited number of values above the detection limit in the control group, making a meaningful comparison of absolute protein levels between the groups impossible. Additionally, proteins with no values above the detection limit in the control group were excluded from the analysis. However, this might lead to an underestimation of the effect of SAH on those proteins, as a rising number of measurable values implies an increasing concentration in CSF. Repeating the study on a broader scale could help to define limit values of the studied proteins to distinguish between physiological and pathological concentrations.

### 3.3. Therapeutic Implications

Since the importance of inflammatory processes in the pathophysiology of SAH is well known, numerous studies testing different anti-inflammatory drugs have been conducted in the past. However, in a recent metanalysis, no influence of these drugs on mortality could be proven, even if some (dapsone and corticosteroids) might improve clinical outcomes [33]. Nevertheless, the FINISHER trial, a randomized controlled trial regarding the use of dexamethasone in SAH patients, is still ongoing and might provide valuable evidence about the therapeutic use of anti-inflammatory medication in this patient group [34].

But even if a non-targeted approach to inflammation remains unsuccessful, the growing understanding of reactive neuroinflammation provides an opportunity to pursue therapeutic interventions more strategically [14].

Recently, growing evidence highlights the interplay between CSF inflammation, blood coagulation, cerebral oedema formation, and CSF dynamics, presenting a specific therapeutic target to improve patient outcomes [18,19]. Although clinical implementation is not yet foreseeable, these new results indicate a real possibility for pharmacologically targeted therapy to influence inflammation.

## 4. Materials and Methods

### 4.1. Inclusion and Exclusion Criteria

In this study, 17 SAH patients were prospectively included. The study was approved by the local ethics committee (Ethikkommission an der Medizinischen Fakultät der Rheinischen Friedrich-Wilhelms-Universität Bonn, Germany; EK 199/08). Written informed consent was obtained from patients or legal representatives. Inclusion requirements were proof of SAH via computed tomography (CT) or lumbar puncture and evidence of an aneurysm as the source of bleeding. As the collection of CSF was part of the study, only SAH patients with acute hydrocephalus requiring an EVD were included. Exclusion criteria were age under 18 years, pregnancy, and refusal to participate in the study.

The control group consisted of 17 patients who received a lumbar drain as part of an aortic surgery. Only controls without cerebrovascular or neurodegenerative diseases or neurological deficits on examination were included. All participants in the control group gave informed consent to take part in the study.

### 4.2. Clinical Management

SAH patients were managed according to the standard operating procedures of Bonn University hospital. After diagnosis of SAH, they received a digital subtraction angiography to identify and further characterize the underlying mechanism. The decision on how to treat the aneurysm (endovascularly vs. open surgically) was made in an interdisciplinary discussion. SAH patients with signs of symptomatic hydrocephalus received an EVD on an emergency basis. After securing the aneurysm, the patients were brought to the neurosurgical intensive care unit (ICU). Clinical severity upon admission was assessed by the WFNS and Hunt and Hess score [35,36]. Radiological extent of SAH in the initial CT scan was assessed by the modified Fisher score [37]. All SAH patients received nimodipine (6 × 60 mg *p*.o.) orally to prevent DCI. According to Vergouwen et al. [8], DCI was defined as secondary neurologic worsening based on a new focal neurological deficit or a decrease of at least two points on the Glasgow Coma Scale (GCS). Other relevant causes for neurological deterioration, such as infection, seizure, and metabolic or electrolyte disturbances, needed to be excluded to diagnose DCI. Seizures were excluded only if observed. However, subclinical seizures were not diagnosed with the help of electric activity monitoring. Neurologic improvement in terms of level of consciousness or degree of muscular strength after induced hypertension was also used as an indicator of DCI. In case of unconsciousness, sedation, or persistent neurological deficit, DCI was also diagnosed radiologically (expanding the clinical definition by Vergouwen et al.) by identifying a new cerebral ischemia or perfusion deficit on CT or magnetic resonance imaging.

Awake SAH patients were closely monitored clinically to assess their neurological status. Unconscious patients were monitored by regular transcranial doppler (TCD) and CT scans as well as advanced neuromonitoring (tissue oxygen saturation probe, intracranial pressure monitoring probe, and microdialysis probe), if appropriate. If DCI (based on clinical or radiological features) was detected, hypertension was induced. In cases where hypertension could not sufficiently improve cerebral perfusion, an endovascular approach was evaluated. All SAH patients stayed in the ICU for a minimum of 14 days. EVDs were left in place until the patient was awake enough to be clinically monitored and mastered an outlet attempt successfully. Outcome at time of discharge and after six months was assessed by the GOS [38]. The GOS was dichotomized into favorable (GOS 4–5) and unfavorable outcome (GOS 1–3).

### 4.3. Sample Collection and Processing

In the control group, 5 mL of CSF was taken from the lumbar drain before the start of aortic surgery.

In the SAH group, CSF samples were taken on day 4 and day 10 after the bleeding from the EVD. Samples were prospectively collected. Due to the planned age and sex matching, a subset of SAH patients corresponding to the control group was selected for the final analyses. CSF mediator levels in the SAH group (taken on days 4 and 10 post-SAH) were compared to the control group’s baseline levels, obtained before the start of surgery.

After sampling, serum and CSF were centrifuged at 2000/min for 10 min, and then, the supernatant was pipetted in a small tube and frozen at −80 °C.

### 4.4. Laboratory Analysis

The immunoassay analysis was performed using the Thermofisher (Waltham, MA, USA) ProcartaPlex Human Neurodegeneration Panel 1 9-plex as well as the ProcartaPlex Hu Cytok./Chemok. Convenience Panel 1 A34-plex and the ProcartaPlex Human Neuroinflammation Panel 6-plex. The assays were performed as instructed by the manufacturer. In short, antibody-coated beads were added to the plate and incubated with the sample. Afterwards, detection antibodies were added and incubated. In the next step, streptavidin–R-phycoerythrin was added and incubated to produce a fluorescence signal, which was analyzed by a Luminex detection system. Luminex analysis and data processing were performed using the xPONENT Software 3.1.871.0.

### 4.5. Choice of Proteins Included in the Analysis

Via the different immunoassays, multiple different proteins were examined, including inflammatory as well as neurodegenerative parameters. Unfortunately, samples were collected in polystyrol containers, so analysis of amyloid was not possible due to potential alteration of results. Therefore, it was decided to focus on neuroinflammatory processes and analyze only chemokines and cytokines. For some of the measured chemokines and cytokines, a huge proportion or even all values were below the detection threshold. Parameters with less than 50% of values in the SAH group or no values in the control group were also excluded.

For a detailed overview of included parameters and their function as well as excluded parameters and the reason for their exclusion, please see Supplementary Tables S1 and S2.

### 4.6. Statistical Analysis

Data analyses and graphing were performed with GraphPad Prism^®^ 10. First, data were distributed by availability of values. If there were more than 50% of the values unavailable in the control group (this was the case when the concentration of the tested substance was below the detection threshold), the number of measurable values was compared via Fisher’s exact test. *p*-values below 0.05 were considered statistically significant. If there were more than 50% of values available for analysis, the sample was tested for normality using the Shapiro–Wilk test. If *p* was greater than 0.05, normality was assumed. In case of normality, unpaired (for SAH vs. control) or paired t-tests (for SAH day 4 vs. SAH day 10) were used. If normal distribution could not be confirmed, the Mann–Whitney test was used for unpaired groups (SAH vs. control) and Wilcoxon test was used for paired groups (SAH day 4 vs. SAH day 10). Since not all datasets were normally distributed, the median and 95% CI are reported. *p*-values below 0.05 were considered significant. Because of the small sample size, outliers were not removed.

## 5. Conclusions

Our data demonstrate the progression of numerous inflammatory parameters in CSF after SAH compared to an age- and sex-matched control group. The extent of reactive neuroinflammation should not be underestimated. To develop effective therapeutic strategies for preventing secondary complications, a broader understanding is essential.

## Figures and Tables

**Table 1:** Demographic characteristics of SAH patients.

Parameter	SAH (*n* = 17)
Female sex	14
Median age	56 (95% CI 49–67 years)
**Clinical condition at admission**	
Hunt and Hess 1–3	10
Hunt and Hess 4–5	7
WFNS I–III	5
WFNS IV–V	12
**Radiological features (modified Fisher)**	
Intracerebral or intraventricular hemorrhage	13
Thick subarachnoid blood clot	4
**Aneurysm location**	
Anterior circulation	13
Posterior circulation	4
**Treatment**	
Clipping	4
Endovascular	13
**Complications**	
DCI	7
Shunt dependency	6
**Outcome at time of discharge**	
Unfavorable (GOS 1–3)	7
Favorable (GOS 4–5)	10
**Outcome after six months**	
Unfavorable (GOS 1–3)	8
Favorable (GOS 4–5)	5
Lost to follow-up	4

## Data Availability

The raw data supporting the conclusions of this article will be made available by the authors on request.

## Supplementary Materials

The following supporting information can be downloaded at: https://www.mdpi.com/article/10.3390/ijms26031302/s1. References [39,40,41,42,43,44,45,46,47,48,49,50,51,52,53,54,55,56,57,58,59,60,61,62,63,64,65] are cited in Supplementary Materials.

## Abbreviations

Aneurysmal subarachnoid hemorrhage	SAH
Early brain injury	EBI
Delayed cerebral ischemia	DCI
Cerebrospinal fluid	CSF
Central nervous system	CNS
World Federation of Neurosurgical Societies score	WFNS
Glasgow Outcome Scale	GOS
Interleukin-1-receptor antagonist	IL-1-RA
Interleukin-6	IL-6
Interleukin-17a	IL-17a
Growth-regulated protein alpha	GRO-α
C-X-C motif chemokine ligand 1	CXCL1
Interleukin-1α	IL-1α
Interleukin-10	IL-10
Interleukin-21	IL-21
Interleukin-9	IL-9
Monocyte Chemoattractant Protein-1	MCP-1
C–C motif chemokine ligand 2	CCL2
C-X-C motif ligand 13	CXCL-13
Eotaxin-1 (CC-chemokine ligand 11)	CCL11
IFN-gamma-inducible protein 10	IP-10
C-X-C motif chemokine ligand 10	CXCL-10
Interleukin-8	IL-8
C-X-C motif chemokine ligand 8	CXCL8
Macrophage migration inhibitory factor	MIF
Macrophage inflammatory protein-1α	MIP-1α
C–C motif chemokine ligand 3	CCL3
Macrophage inflammatory protein-1β	MIP-1β
C–C motif chemokine ligand 4	CCL4
Stromal cell-derived factor 1α	SDF-1 α
C-X-C motif chemokine ligand 12	CXCL12
Interleukin-2	IL-2)
Triggering receptor expressed on myeloid cells-2	TREM-2
Traumatic brain injury	TBI
External ventricular drain	EVD
Computed tomography	CT
Intensive care unit	ICU
Glasgow Coma Scale	GCS
Transcranial doppler	TCD
Confidence intervals	CI
