# Supplementary material for: Mapping Inflammatory Markers in Cerebrospinal Fluid Following Aneurysmal Subarachnoid Hemorrhage: An Age- and Sex-Matched Analysis
Source: Int J Mol Sci. 2025 Feb 3;26(3):1302. doi: 10.3390/ijms26031302 (PMC11818219; doi:10.3390/ijms26031302)
Supplement: Supplementary file 1 [file ijms-26-01302-s001.zip › ijms-3354983-supplementary.pdf]

Supplementary Materials

1. Supplementary Table S1: Summary of key properties and function of the analysed cytokines and chemokines
2. Supplementary Table S2: Summary of excluded proteins and the reason why they were excluded from analysis.
3. Supplementary Table S3: Age- and sex-matched pairs of control and SAH patients.
4. Supplementary Figure S1: Comparison of the number of available values for IL-4, IL-1-RA, IL-6, IL-17a, GRO-α, IL-1α, IL-10, IL-21 and IL-9 between the control and SAH group on both days.
5. Subgroup analysis

Supplementary Table S1: Summary of key properties and function of the analysed cytokines and chemokines.

Protein	Function
**Cytokines**	
Interleukin-1 α (IL-1 α)	Pro-inflammatory function [39]
Interleukin-1β (IL-1β)	Induction of inflammation and fever, involved in neurodegeneration [40]
Interleukin-1-receptor antagonist (IL1-RA)	Downregulation of inflammatory processes [39,40]
Interleukin -10 (IL-10)	Immunosuppression, resolves inflammation and promotes wound repair [41]
Interleukin-17a (IL-17a)	Expressed by TH17 cells, proinflammatory function, involved in neuroinflammatory, infectious and psychiatric diseases as well as ischemic stroke [42]
Interleukin 2 (IL-2)	Modulates regulatory t-cells and can therefore regulate autoimmune processes and neuroinflammation [43,44]
Interleukin-21 (IL-21)	Regulation of multiple immune cells, can increase and inhibit immune responses, involved in multiple autoimmune diseases and multiple sclerosis [45]
Interleukin-6 (IL-6)	Pro-inflammatory and neurotrophic, involved in neurodegenerative and neuroinflammatory diseases as well as in physiological function of neurons and glia cells [46]
Interleukin-9 (IL-9)	Pro- and anti-inflammatory properties depending on the cell-type, seems to be involved in neuroinflammatory processes [47–49]
Triggering receptor expressed on myeloid cells-2 (TREM-2)	Modulation of microglia, mutations/deficiency are associated with neurodegenerative diseases and progression of certain brain tumors [50,51]
Macrophage migration inhibitory factor (MIF)	Immune-modulation, modulation of neuroprotection and neuropasticity, involved in neurodegenerative, neuroinflammatory and psychiatric diseases as well as ischemic stroke [52]
**Chemokines**	
Eotaxin-1 (CC-chemokine ligand 11/CCL-11)	Attraction of eosinophiles, involved in multiple psychiatric, neurodegenerative and neuroinflammatory diseases [53]
Growth-regulated protein alpha (GRO-α)/ C-X-C motif chemokine ligand 1 (CXCL-1)	Attraction of immune cells, involved in prenatal brain development as well as neurodegenerative and neuroinflammatory diseases and ischemic stroke [54]
C-X-C motif ligand 13 (CXCL-13)	Attraction of b-cells and $CD4^+$ t-follicular helper (Tfh) cells, involved in primary central nervous system lymphoma and neuroinflammation [55]
Interleukin 8 (IL-8)/ C-X-C motif chemokine ligand 8 (CXCL-8)	Attraction of neutrophile granulocytes, involved in neurodegenerative diseases and tumor angiogenesis [56,57]

IFN-gamma-inducible protein 10 (IP-10)/ C-X-C motif chemokine ligand 10 (CXCL-10)	Attraction of t-cells and natural killer cells, involved in neurodegenerative and neuroinflammatory diseases [58]
Monocyte Chemoattractant Protein-1 (MCP-1)/ C–C motif chemokine ligand 2 (CCL2)	Attraction of monocytes and macrophages, involved in neuroinflammatory diseases [59,60]
Macrophage inflammatory protein-1α (MIP-1α))/ C–C motif chemokine ligand 3 (CCL-3)	Attraction of macrophages and granulocytes, involved in neuroinflammation [61,62]
Macrophage inflammatory protein-1β (MIP-1β)/ C–C motif chemokine ligand 4 (CCL4)	Attraction of monocytes, t-cells, dendritic cells, natural killer cells, involved in neuroinflammation [63,64]
Stromal cell-derived factor 1α (SDF-1 α)/ C-X-C motif chemokine ligand 12 (CXCL-12)	Attraction of lymphocytes and monocytes, involved in primary brain tumors , stroke and neuroinflammation [65]

Supplementary Table S2: Summary of excluded proteins and the reason why they were excluded from analysis.

Protein	Reason for Exclusion
Vascular Endothelial Growth Factor C (VEGF C)	No values available in the SAH group at both time points
Angiopoietin 2	No values available in the SAH group at both time points
Granulocyte macrophage-colony stimulating factor (GM-CSF)	No values available in the SAH group at both time points
Interferon γ (IFN-γ)	No values available in the SAH group at both time points
Interleukin 12p70 (IL-12p70)	No values available in the SAH group at both time points
Interleukin 13 (IL-13)	No values available in the SAH group at both time points
Interleukin 15 (IL-15)	No values available in the SAH group at both time points
Interleukin 18 (IL-18)	Less than 50% of values available in all groups
Interleukin 22 (IL-22)	Only one value available at day 4 after SAH, no values available in the other groups
Interleukin 23 (IL-23)	No values available in the SAH group at both time points
Interleukin 27 (IL-27)	No values available in the SAH group at both time points
Interleukin 31 (IL-31)	No values available in the SAH group at both time points
Interleukin 5 (IL-5)	Only one value available at day 4 after SAH, no values available in the other groups
Interleukin 7 (IL-7)	No values available in the control group
Tumor necrosis factor α (TNF-α)	Less than 50% of values available in all groups
Tumor necrosis factor β (TNF-β)	No values available in the SAH group at both time points
Kalikrein-6	No values available in the control group and less than 50% of values available in SAH group at both time points
Amyloid beta 1-40	Not usable, as CSF was sampled in polystyrol containers
Amyloid beta 1-42	Not usable, as CSF was sampled in polystyrol containers
Tau-total	Focus on neuroinflammation, not neurodegeneration
Neural cell adhesion molecule 1 (NCAM-1)	Focus on neuroinflammation with cytokines and chemokines
Tau-pT181	Focus on neuroinflammation, not neurodegeneration
TAR DNA-binding protein 43 (TDP-43)	No values available in the control group
Fibroblast growth factor 21 (FGF-21)	No values available in the SAH group at both time points
Fibroblast growth factor 22 (FGF-22)	No values available in the SAH group at both time points
Interleukin 34 (IL-34)	Less than 50% of values available in all groups
Receptor for advanced glycation endproducts (RAGE)	Focus on neuroinflammation with cytokines and chemokines
Chitinase-3-like protein 1 (CHI3L1)/ YKL-40	Significantly higher values in the control group which could not be explained by demographic factors or concomitant illnesses, so a measuring error is suspected

Fas ligand (Fas L)	No values available in SAH group at both time points
Interleukin 9 (IL-9)	No values available in SAH group at both time points
Vascular Endothelial Growth Factor A (VEGF A)	No values available in SAH group at both time points
Angiopoietin 1	Less than 50% of values available in all groups
Neurogranin	Focus on neuroinflammation with cytokines and chemokines
C–C motif chemokine ligand 5 (CCL5/ regulated upon activation, normal T cell expressed and secreted (RANTES)	Less than 50% of values available in the control group and on day 10 after SAH
Interleukin-4 (IL-4)	No values available in the control group
Interferon-α (IFN-α)	No values available in the control group
Interleukin 1β (IL-1β)	Less than 50% of values available in all groups

Supplementary Table S3: Age- and sex-matched pairs of control and SAH patients.

Patient number	Control sex	Control age	SAH sex	SAH age
1	0	74	0	74
2	1	73	1	67
3	1	69	1	49
4	1	78	1	60
5	1	40	1	54
6	0	38	0	39
7	1	76	1	70
8	1	57	1	68
9	1	74	1	55
10	1	71	1	45
11	1	64	1	56
12	1	49	1	44
13	1	66	1	52
14	1	65	1	43
15	1	66	1	71
16	1	61	1	56
17	0	58	0	58

Supplementary Figure S1:

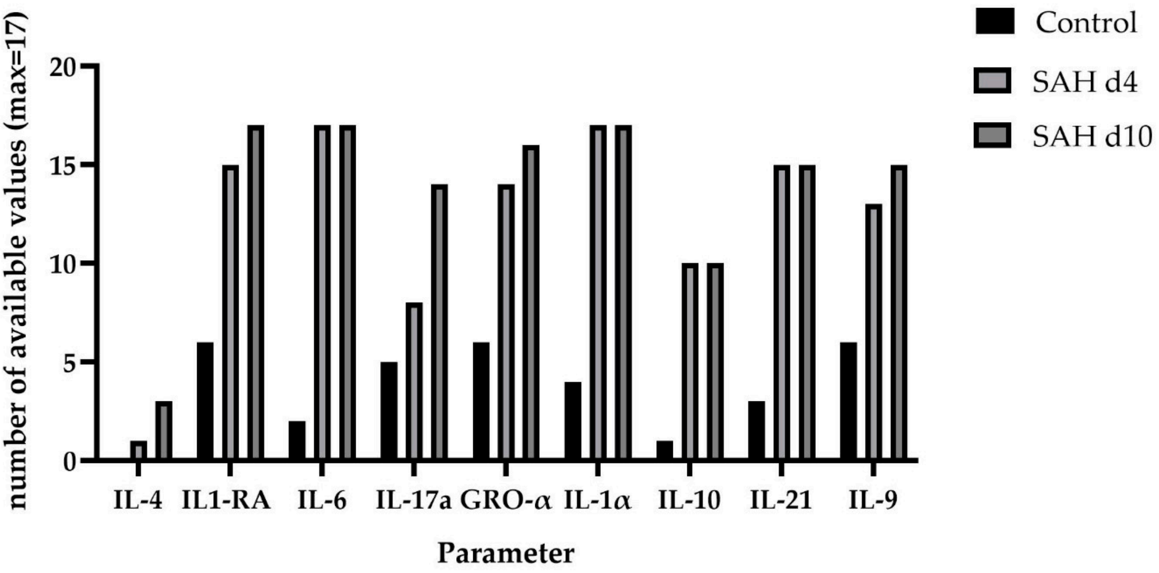

Supplementary Figure S1: Comparison of the number of available values for IL-4, IL-1-RA, IL-6, IL-17a, GRO-α, IL-1α, IL-10, IL-21 and IL-9 between the control and SAH group on both days. For these parameters, only a few values were above the detection limit in the control group, making a comparison of absolute values difficult. However, no statistically significant difference could be detected between control and SAH patients at both time points.

Subgroup analyses

Subgroup analyses were conducted for the following demographic characteristics. Since all parameters were not normally distributed, data was dichotomized and Mann-Whitney test was used to compare the groups. P-values below 0.05 were considered statistically significant.

Hunt&Hess: There were no significant differences between Hunt&Hess 1-3 and Hunt&Hess 4-5 neither on day 4 nor on day 10 after SAH.

Age: There were no significant differences comparing the age groups below sixty and above sixty years neither on day 4 nor on day 10 after SAH.

Sex: There were no significant differences comparing sex neither on day 4 nor on day 10 after SAH.

DCI: There were no significant differences comparing DCI neither on day 4 nor on day 10 after SAH.

Shunt dependency: There were no significant differences comparing Shunt dependency neither on day 4 nor on day 10 after SAH.

To conclude, no significant differences were found between the different groups at both time points, which might be due to the small sample size.

References

39. Dinarello, C.A. Overview of the IL-1 Family in Innate Inflammation and Acquired Immunity. *Immunol Rev* **2018**, *281*, 8–27, doi:10.1111/imr.12621.

40. Boraschi, D.; Italiani, P.; Migliorini, P.; Bossù, P. Cause or Consequence? The Role of IL-1 Family Cytokines and Receptors in Neuroinflammatory and Neurodegenerative Diseases. *Front. Immunol.* **2023**, *14*, doi:10.3389/fimmu.2023.1128190.

41. Burmeister, A.R.; Marriott, I. The Interleukin-10 Family of Cytokines and Their Role in the CNS. *Front Cell Neurosci* **2018**, *12*, 458, doi:10.3389/fncel.2018.00458.

42. Waisman, A.; Hauptmann, J.; Regen, T. The Role of IL-17 in CNS Diseases. *Acta Neuropathol* **2015**, *129*, 625–637, doi:10.1007/s00401-015-1402-7.

43. Lykhopiy, V.; Malviya, V.; Humblet-Baron, S.; Schlenner, S.M. IL-2 Immunotherapy for Targeting Regulatory T Cells in Autoimmunity. *Genes Immun* **2023**, *24*, 248–262, doi:10.1038/s41435-023-00221-y.

44. Yshii, L.; Pasciuto, E.; Bielefeld, P.; Mascali, L.; Lemaitre, P.; Marino, M.; Dooley, J.; Kouser, L.; Verschoren, S.; Lagou, V.; et al. Astrocyte-Targeted Gene Delivery of Interleukin 2 Specifically Increases Brain-Resident Regulatory T Cell Numbers and Protects against Pathological Neuroinflammation. *Nat Immunol* **2022**, *23*, 878–891, doi:10.1038/s41590-022-01208-z.

45. Shbeer, A.M.; Ahmed Robadi, I. The Role of Interleukin-21 in Autoimmune Diseases: Mechanisms, Therapeutic Implications, and Future Directions. *Cytokine* **2024**, *173*, 156437, doi:10.1016/j.cyto.2023.156437.

46. Rothaug, M.; Becker-Pauly, C.; Rose-John, S. The Role of Interleukin-6 Signaling in Nervous Tissue. *Biochimica et Biophysica Acta (BBA) - Molecular Cell Research* **2016**, *1863*, 1218–1227, doi:10.1016/j.bbamcr.2016.03.018.

47. Pajulas, A.; Zhang, J.; Kaplan, M.H. The World According to IL-9. *The Journal of Immunology* **2023**, *211*, 7–14, doi:10.4049/jimmunol.2300094.

48. Li, H.; Nourbakhsh, B.; Ciric, B.; Zhang, G.-X.; Rostami, A. Neutralization of IL-9 Ameliorates Experimental Autoimmune Encephalomyelitis by Decreasing the Effector T Cell Population. *The Journal of Immunology* **2010**, *185*, 4095–4100, doi:10.4049/jimmunol.1000986.

49. Ding, X.; Cao, F.; Cui, L.; Ciric, B.; Zhang, G.-X.; Rostami, A. IL-9 Signaling Affects Central Nervous System Resident Cells during Inflammatory Stimuli. *Experimental and Molecular Pathology* **2015**, *99*, 570–574, doi:10.1016/j.yexmp.2015.07.010.

50. Painter, M.M.; Atagi, Y.; Liu, C.-C.; Rademakers, R.; Xu, H.; Fryer, J.D.; Bu, G. TREM2 in CNS Homeostasis and Neurodegenerative Disease. *Molecular Neurodegeneration* **2015**, *10*, 43, doi:10.1186/s13024-015-0040-9.

51. Zhong, J.; Xing, X.; Gao, Y.; Pei, L.; Lu, C.; Sun, H.; Lai, Y.; Du, K.; Xiao, F.; Yang, Y.; et al. Distinct Roles of TREM2 in Central Nervous System Cancers and Peripheral Cancers. *Cancer Cell* **2024**, *42*, 968-984.e9, doi:10.1016/j.ccell.2024.05.001.

52. Zhang, Y.; Yu, Z.; Ye, N.; Zhen, X. Macrophage Migration Inhibitory Factor (MIF) in CNS Diseases: Functional Regulation and Potential Therapeutic Indication. *Fundamental Research* **2024**, *4*, 1375–1388, doi:10.1016/j.fmre.2023.05.008.

53. Ivanovska, M.; Abdi, Z.; Murdjeva, M.; Macedo, D.; Maes, A.; Maes, M. CCL-11 or Eotaxin-1: An Immune Marker for Ageing and Accelerated Ageing in Neuro-Psychiatric Disorders. *Pharmaceuticals (Basel)* **2020**, *13*, 230, doi:10.3390/ph13090230.

54. Korbecki, J.; Gąssowska-Dobrowolska, M.; Wójcik, J.; Szatkowska, I.; Barczak, K.; Chlubek, M.; Baranowska-Bosiacka, I. The Importance of CXCL1 in Physiology and Noncancerous Diseases of Bone, Bone Marrow, Muscle and the Nervous System. *Int J Mol Sci* **2022**, *23*, 4205, doi:10.3390/ijms23084205.

55. Irani, D.N. Regulated Production of CXCL13 within the Central Nervous System. *J Clin Cell Immunol* **2016**, *7*, 460, doi:10.4172/2155-9899.1000460.

56. Righi, D.; Manco, C.; Pardini, M.; Stufano, A.; Schino, V.; Pelagotti, V.; Massa, F.; Stefano, N.D.; Plantone, D. Investigating Interleukin-8 in Alzheimer's Disease: A Comprehensive Review. *Journal of Alzheimer's Disease* **2024**, 13872877241298973, doi:10.1177/13872877241298973.
57. Matsushima, K.; Yang, D.; Oppenheim, J.J. Interleukin-8: An Evolving Chemokine. *Cytokine* **2022**, *153*, 155828, doi:10.1016/j.cyto.2022.155828.
58. Michlmayr, D.; McKimmie, C.S. Role of CXCL10 in Central Nervous System Inflammation. *IJICMR* **2014**, *6*, 1–18, doi:10.2147/IJICMR.S35953.
59. Conductier, G.; Blondeau, N.; Guyon, A.; Nahon, J.-L.; Rovère, C. The Role of Monocyte Chemoattractant Protein MCP1/CCL2 in Neuroinflammatory Diseases. *Journal of Neuroimmunology* **2010**, *224*, 93–100, doi:10.1016/j.jneuroim.2010.05.010.
60. Deshmane, S.L.; Kremlev, S.; Amini, S.; Sawaya, B.E. Monocyte Chemoattractant Protein-1 (MCP-1): An Overview. *J Interferon Cytokine Res* **2009**, *29*, 313–326, doi:10.1089/jir.2008.0027.
61. Bhavsar, I.; Miller, C.S.; Al-Sabbagh, M. Macrophage Inflammatory Protein-1 Alpha (MIP-1 Alpha)/CCL3: As a Biomarker. *General Methods in Biomarker Research and their Applications* **2015**, 223–249, doi:10.1007/978-94-007-7696-8_27.
62. Puthenparampil, M.; Stropparo, E.; Zywicki, S.; Bovis, F.; Cazzola, C.; Federle, L.; Grassivaro, F.; Rinaldi, F.; Perini, P.; Sormani, M.P.; et al. Wide Cytokine Analysis in Cerebrospinal Fluid at Diagnosis Identified CCL-3 as a Possible Prognostic Factor for Multiple Sclerosis. *Front. Immunol.* **2020**, *11*, doi:10.3389/fimmu.2020.00174.
63. Szczuciński, A.; Losy, J. Chemokines and Chemokine Receptors in Multiple Sclerosis. Potential Targets for New Therapies. *Acta Neurologica Scandinavica* **2007**, *115*, 137–146, doi:10.1111/j.1600-0404.2006.00749.x.
64. Menten, P.; Wuyts, A.; Van Damme, J. Macrophage Inflammatory Protein-1. *Cytokine & Growth Factor Reviews* **2002**, *13*, 455–481, doi:10.1016/S1359-6101(02)00045-X.
65. Li, M.; Ransohoff, R.M. Multiple Roles of Chemokine CXCL12 in the Central Nervous System: A Migration from Immunology to Neurobiology. *Prog Neurobiol* **2008**, *84*, 116–131, doi:10.1016/j.pneurobio.2007.11.003.
